# Analysis of Muscle Strength and Electromyographic Activity during Different Deadlift Positions

**DOI:** 10.3390/muscles2020016

**Published:** 2023-05-08

**Authors:** Vinícius Marques Moreira, Leonardo Coelho Rabello de Lima, Arnaldo Luis Mortatti, Thiago Mattos Frota de Souza, Fernando Vitor Lima, Saulo Fernandes Melo Oliveira, Christian Emmanuel Torres Cabido, Felipe J. Aidar, Manoel da Cunha Costa, Thiago Pires, Tatiana Acioli, Rogério César Fermino, Cláudio Oliveira Assumpção, Túlio Banja

**Affiliations:** 1 Institute of Physical Education and Sports, Federal University of Ceará, Fortaleza 60020-181, Brazil; 2 School of Physical Education and Sport of Ribeirão Preto, University of São Paulo, Ribeirão Preto 14040-900, Brazil; 3 Graduate Program in Physical Education, Federal University of Rio Grande do Norte, Natal 59078-970, Brazil; 4 Applied Kinesiology Laboratory—LCA, School of Physical Education, University of Campinas, Campinas 13083-959, Brazil; 5 Weight Training Laboratory, School of Physical Education, Physiotherapy and Occupational Therapy, Federal University of Minas Gerais, Belo Horizonte 31270-901, Brazil; 6 Physical Education Graduate Program, Vitória Academic Center, Federal University of Pernambuco, Recife 50800-220, Brazil; 7 Postgraduate Program in Physical Education Program in Physical Education, Department of Physical Education, Laboratory of the Physical Exercise Research Group: Health and Human Performance, Federal University of Maranhão, São Luís 65080-805, Brazil; 8 Department of Physical Education, Federal University of Sergipe, São Cristóvão 49100-000, Brazil; 9 Graduate Program Association of Physical Education, Federal University of Paraíba, João Pessoa 58051-900, Brazil; 10 Human Performance Laboratory, State University of Pernambuco, Recife 50100-010, Brazil; 11 UNIFBV—University Center, Recife 51200-060, Brazil; 12 Research Group in Environment, Physical Activity, and Health, Postgraduate Program in Physical Education, Federal University of Technology—Paraná, Curitiba 80230-901, Brazil; 13 Postgraduate Program in Physical Education, Federal University of Paraná, Curitiba 81530-900, Brazil; 14 Exercise Science, Health and Human Performance Research Group, Department of Sport Sciences, Institute of Health Sciences, Federal University of Triângulo Mineiro, Uberaba 38025-350, Brazil

**Keywords:** isometric strength, muscle activity, electromyography

## Abstract

The aim of the study was to analyze muscle activation in the three positions of the deadlift (DL). Twenty male participants (33.4 ± 3.9 years; 42.2 ± 9.1 months of experience with DL; 91.0 ± 14.8 kg; and 1.78 ± 0.06 m) pulled a bar through isometric actions in three DL positions: lift-off, mid-pull, and lockout. Isometric strength, knee angle, and activation of the rectus femoris (RF), biceps femoris (BF), lateral gastrocnemius (GAL), and erector spinae (ERE) muscles were collected. The analysis of variance showed that the maximum isometric force presented differences between the positions (*p* = 0.001; η^2^ = 0.973) considered large with higher values at the mid-pull position. Interactions were found between muscles and position (*p* = 0.001; η^2^ = 0.527) considered large. The RF and ERE showed greater activation in the lift-off position, while in the mid-pull position, there was greater activation of the BF and GAL muscles. The DL positions produce different activations in the bi-articular and uni-articular muscles. The lift-off requires more activation from the RF and ERE positions. The mid-pull position, despite generating greater force, presented greater activations in the BF and GAL. The ERE showed higher activations as the external torque was greater.

## 1. Introduction

Powerlifting has been growing in popularity in recent years and comprises squat, bench press and deadlift (DL) exercises [1]. Specifically, DL has been used as a strengthening exercise in strength training regimens of recreational athletes aiming to improve lower limb, hip, upper limb, and trunk strength [2].

Deadlifting consists of pulling a bar from the floor with both hands by extending the knees, hips, and trunk while maintaining constant elbow extension. By the end of the DL movement, knees and hips must be in a locked position and the scapulae, retracted [3]. During DLs, the quadriceps and hamstrings muscles are activated and act to promote tension in the hip and knee joints. Gastrocnemii muscles act in the ankle and knee joints while ERE muscles are responsible for maintaining trunk stability and extension throughout the movement. Due to their biarticular characteristic, the RF and BF (as a component of the hamstring muscles) are activating during all phases of the DL movement [4]. Hence, as joint angles change the activation of each of these muscles and their contribution to force production in the hip and knees are affected [5]. Synchronized changes in muscle length and momentum arm result in torque variations, which are mostly determined by changes in the length-tension curve [6], and this appears to differ in distinct muscles.

Some authors [7,8] propose that there are three main positions during the DL: (1) lift-off when force is applied to the bar to pull it from the floor; (2) mid-pull, when the bar is located immediately above the knees; and (3) lockout, when the lifter’s trunk reaches the vertical position, with the bar positioned at its highest point during the DL. Other authors investigated DL performance in these positions [9,10,11] and there is a consensus that mid-pull is the most challenging DL position. The greatest force values assessed during DL are observed at the mid-pull position, with the same group later suggesting that this might be due to participants feeling more comfortable in this position [12].

A recent systematic review investigating electromyographic (EMG) records points out that the BF is the most frequently investigated muscle during DL, followed by the gluteus maximus (GLU), vastus lateralis (VL), and the ERE [12]. The semitendinous (ST) and RF follow-up, with the vastus medialis (VM), external oblique (EO), gastrocnemius medialis (GM), and lateralis (GAL) [10] also being frequently investigated. A study [10] compared muscle activation between two variations of DL exercise (conventional vs. sumo) with knee joint angles ranging between 30° and 90°. It was found that the activations of quadriceps and ERE muscles were greater when the knee joint was more flexed, while hamstring and gastrocnemii muscles were most activated as the knee flexion angle decreased during the eccentric phase [13]. The activation of BF peaked at the onset of concentric contractions during DL exercise while GM was more activated close to the lockout position. Similarly, another study [4] showed that activation of the RF and GLU activation was greater during conventional DL exercise compared to the sumo and Romanian DL variations. Additionally, the ERE are the most activated muscles during DL regardless of load variations [14].

The aforementioned studies investigated different muscles according to their respective hypotheses. However, there is a lack of studies investigating the activation of biarticular muscles during different positions of the DL exercise, which might exhibit changes in muscle activation due to changes in joint angles. To the best of our knowledge, Edington et al. [15] were the only ones to investigate muscle activation during DL exercise performed isometrically in three different positions, and the only muscle investigated was the BF. Investigating which biarticular muscle is activated the most during different positions can provide valuable information regarding the contributions of these muscles across the entire range of motion of DL exercise. The information about the muscles that act on more than one joint may bring relevant information, especially when these muscles act simultaneously. This knowledge could be applied in rehabilitation programs when the intention is to strengthen specific muscle groups or improve strength in a given posture during DL execution.

Therefore, the aim of this study was to compare the activation of the main biarticular muscles during three key positions of the DL. Since previous studies report differences in muscle activation during concentric and eccentric DL actions, we hypothesized that there will be different activation patterns of the investigated muscles in each of the three positions.

## 2. Results

**Figure 1:**
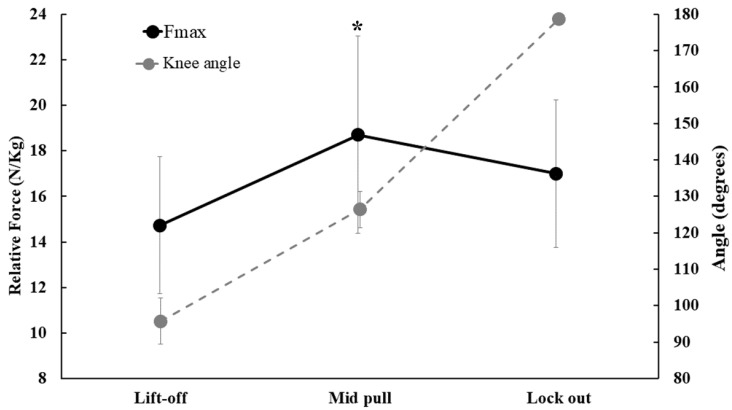
Muscle strength and knee joint range in the three DL positions. * Significant difference in relative strength for the lift-off position (*p* < 0.05).

The maximal isometric strength values showed differences between the three DL positions [F (1, 20); 539.204; *p* = 0.001], with an effect size η^2^ = 0.973 considered large. The lift-off (14.77 ± 3.0 N/kg) and mid-pull (18.67 ± 4.34 N/kg) positions showed the lowest and highest relative strengths, respectively. The posthoc test showed that the lift-off position was different from the mid-pull (*p* = 0.001), with no differences between the lift-off and lock-out positions (*p* = 0.084) and no differences between the mid-pull and lock-out positions (*p* = 0.321). Figure 1 shows the results of the relative strength and angle of the knee joint.

**Figure 2:**
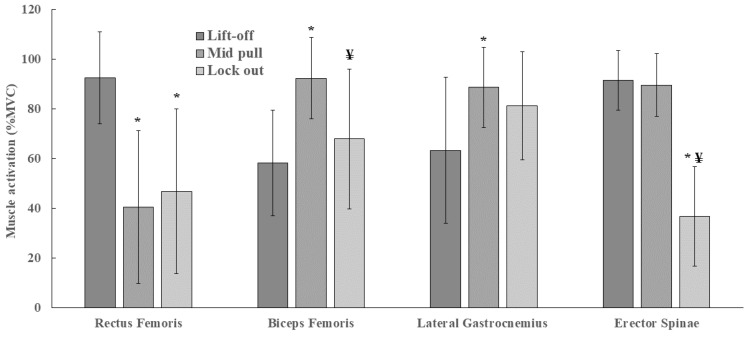
Muscle activity in relation to DL positions. * Significant difference (*p* < 0.05) for the lift-off position. ¥ Significant difference (*p* < 0.05) for the mid-pull position.

Regarding the electromyographic activity of the muscles in the 3 evaluated positions, interactions were found between muscles and position [F (3.672, 69.762); 21.132; *p* = 0.001], with an effect size η^2^ = 0.527 considered large. In relation to each muscle, the activation values for the RF in the lift-off, mid-pull, and lock-out positions were 92.46 ± 18.42, 40.50 ± 30.79, and 46.85 ± 33.19% MVC, respectively. The posthoc test showed that the RF muscle presented differences between the lift-off and mid-pull positions (*p* = 0.001) and between the lift-off and lock-out positions (*p* = 0.001) but with no differences between the mid-pull and lock-out positions (*p* = 0.998). The activation values for BF in the lift-off, mid-pull, and lock-out positions were 58.22 ± 21.33, 92.27 ± 16.45, and 67.94 ± 28.08% MVC, respectively. BF showed differences between the lift-off and mid-pull positions (*p* = 0.001), with no differences between the lift-off and lock-out positions (*p* = 0.922), with differences between the mid-pull and lock-out positions (*p* = 0.015). The activation values for GAL in the lift-off, mid-pull, and lock-out positions were 63.32 ± 29.31, 88.58 ± 16.18, and 81.23 ± 21.73% MVC, respectively. GAL showed differences between the lift-off and mid-pull positions (*p* = 0.013) but with no differences between the lift-off and lock-out positions (*p* = 0.107) and the mid-pull and lock-out positions (*p* = 0.849). The activation values for ERE in the lift-off, mid-pull, and lock-out positions were 91.46 ± 11.94, 89.51 ± 12.53, and 36.79 ± 19.90% MVC, respectively. ERE showed differences between the lift-off and lock-out positions (*p* = 0.001) and between the mid-pull and lock-out positions (*p* = 0.001) but with no differences between the lift-off and mid-pull positions (*p* = 0.999). Figure 2 shows the results of the interaction between muscle activation values and DL positions.

## 3. Discussion

The aim of this study was to compare the activation of the main biarticular muscles during three key positions of the DL. The main findings of our study showed that the mid-pull position generated higher strength among the three positions and that RF and ERE muscles were the most active in the lift-off position, while BF and GAL were the most active in the mid-pull position in DL.

Our results agree with [9,16] regarding the higher relative strength in the mid-pull position. According to the authors, it occurred because this position offers a greater advantage in strength production, as the powerful extensor strengths of the quadriceps and hip extensor muscles are used. The strength in this position is also higher than in another DL variation, the sumo [3]. The lock-out position was the second that generated the most strength and the lift-off was the least. It is probably related to the muscle length-tension curve [17] in which the muscle capacity is optimal at approximately half the joint amplitude, being lowest at the beginning and end of this movement.

RF muscle showed higher activation in the lift-off position. These results are similar to those found in [10]. It occurs because the smaller angle of the knee and hip would request higher demand from this muscle, while the hamstring muscles would perform the knee stabilization function. In addition, the RF muscle is also a hip flexor and would only need to act on the knee joint in this position [4]. However, Reference [10] reported that the other portions of the quadriceps group (vastus lateralis and vastus medialis) that do not act on the hip joint present higher activations than RF in this position. Although in our study we did not evaluate the vastus lateralis muscle, other studies have shown higher activations of this muscle compared to RF in the initial phase of DL [14]. RF showed the lowest activation in the mid-pull position, with differences for the initial position. This result is similar to that found by [10], and a decreased activity in other quadriceps muscles has also been reported [13,14]. The decrease in activation in this position of muscles of the quadriceps group would occur by changing the position of the hip in extension [5,15]. Hip extension action in this position is more evident and will be discussed next when analyzing the BF muscle. RF in the lock-out position showed the second-highest activation. Our data disagree with the results by [10], who found higher RF activity as they reached this position. It probably occurred in the aforementioned study because they were evaluated by dynamic contractions, as the isometric action presents higher activation than concentric and eccentric actions.

BF showed less activation in the lift-off position compared to the other positions. It occurred when the knee and hip joint were more flexed in this position and BF was more elongated. Therefore, in an unfavorable position due to the length-tension relationship, especially in relation to the hip joint. According to [5], some motor units in this position might not be active because they were in a position where they could no longer produce active strength. According to this author, it would be common in the hamstring muscles. BF showed higher activation in the mid-pull position. These results agree with other studies [4,10,13,14,15]. This increased activation is the result of a position in which BF is acting as a hip extensor at an optimal angle. Another point is that the amplitude of the knee in this position is closer to maximum extension than the hip joint, generating a higher possibility of tension in BF as the hip reaches maximum extension. The maximum torque in isometric hip extensions is higher when BF is in a position more elongated by knee extension [18] which would correspond to the mid-pull position.

BF showed the second-highest activation in the lock-out position. Our results agree with [14,15]. It occurred due to the higher request of muscle fibers to sustain hip extension without flexing the knee when the hip reaches 180°. The hamstring tendon is very close to the knee joint axis when the knee is fully extended, providing a poor leverage arm for knee flexion. In addition, the muscle is elongated in both joints. The interaction of these two factors may have caused the observed decrease in EMG activity [5]. Furthermore, some authors have argued that BF is selectively recruited to deal with hip and knee joint movement during hip extension exercises [19,20].

EMG amplitude of RF during isometric knee extensions was significantly higher when the knee and hip joint were maximally flexed (lift-off), decreasing as these joints extended (mid-pull), with the reverse effect occurring on the BF muscle, indicating that the RF activation pattern was opposite to BF. This behavior was reported during isometric exercise in the leg extension machine for 90°, 120°, and 150° [21]. These authors found similar behaviors to our study, but they did not verify the influence of the joint amplitude in RF activation. This alternation of higher muscle activity between RF and BF could be related to the phenomenon of reciprocal inhibition between antagonist muscles [22].

GAL showed higher activation during mid-pull than the positions Lift off and lock-out, which may have occurred because this position demands higher torque in the plantar flexor muscles and, consequently, higher EMG activity [4,15]. The results found by [10] agree with our findings, considering that these authors reported higher GAL activation in a position that would be close to mid-pull. Some participants performed plantar flexion during the lock-out position. It could explain the similar activation of this muscle in this position compared to the mid-pull. However, plantar flexion during the lock-out position is not part of the powerlifting movement, as this action occurs in the clean and snatch movements, which are related to another form of lifting, weightlifting.

ERE showed the highest activations in the lift-off and mid-pull positions. This result is similar to findings reported by other studies [4,13,14]. According to [23], the increase in activation in this muscle contributed to a decrease in the lumbar spine shear force, decreasing the external load on DL. The lift-off position in DL, using the powerlift posture, generates greater lumbar spine shear force. However, another study [15] found that the variation in the choice of body positioning at this initial moment does not modify ERE activation. Thus, the activation of this muscle during the lift-off and mid-pull positions would be related to the higher demand in the isometric action of the trunk. In these two positions, leaning the trunk forward results in higher spinal flexion torque generated by the barbell. Therefore, ERE requires higher activation and higher strength to avoid trunk flexion, reducing shear [23]. It could explain the decrease in ERE activity in the lock-out position, as the amount of torque in DL depends on the distance from the center of mass of the upper body and the load from the pivot point, consisting of the hips [24].

Some limitations should be considered in this study. Although all participants are familiar with DL, isometric exercise is not part of their training routine. Another point in question concerns the posture of the participants in the lift-off position. Despite attempts to adjust the posture, some participants adopted the Weightlifting posture, which could especially influence the activation of RF and BF muscles in the lift-off.

In conclusion, our study revealed that the different DL positions resulted in varying activations in biarticular and uniarticular muscles. Specifically, the lift-off position required greater activation of the RF and ER muscles, whereas the mid-pull position only showed increased activations in the LF and GAL muscles despite generating greater strength. Additionally, greater external torque in the ERE position resulted in higher activations of the ERE muscle.

Notably, RF activation was significantly greater during the lift-off position, when the knee and hip joints were maximally flexed. Conversely, in the mid-pull position, as the hip and knee joints extended, the activation pattern of the RF muscle was opposite to that of the BF, with less activation in the latter.

## 4. Materials and Methods

### 4.1. Participants

The sample was intentional and not probabilistic. The sample size was determined by G*Power v3.1.9.6 software (G*Power, Kiel, Germany) from the effect size of the study by [14]. The effect size of 0.89 (considered a large effect) was adopted, with a statistical power of 99% and a *p*-value = 0.05, resulting in a minimum of 12 participants. Twenty male participants aged 33.4 ± 3.9 years, experience with DL of 42.2 ± 9.1 months, body mass of 91.0 ± 14.8 kg, and height of 1.78 ± 0.06 m. Participants should be recreationally trained in activities that perform DL exercises for at least 12 months to participate in the research, being able to overcome a load equivalent to their body mass in DL. Participant indication, general warm-up, and technique analysis during the execution of this movement were supervised by an experienced trainer. Participants were instructed not to exercise 24 h before any of the sessions. The exclusion criteria were: (1) injury in the last three months and (2) any muscle pain or discomfort that would disable the participant from performing the tests. The individuals, after being previously informed about the purposes of the investigation and the procedures to which they would be submitted, agreed to participate voluntarily in the study and signed a Free and Informed Consent Term following the norms of Resolution 196/96 of the National Council of Health on research involving human beings. The procedures were approved by the local Research Ethics Committee no. 3.290.772.

### 4.2. Experimental Protocol

The test was performed in just one day. The warm-up consisted of performing 4 progressive sets of DL with different loads and intervals [16]. It consisted of 2–3 reps at 35% of 1 repetition maximum (1-RM), followed by a 90-s rest interval; 2–3 reps at 50% 1-RM, followed by a 120-s rest interval; 1–2 reps at 65% 1-RM, followed by 150-s rest interval; and finally, 1 rep at 75% 1-RM, followed by a 180-s rest interval. Warm-up loads were determined using each participant’s 100% 1-RM recordings. The knee joint angle was measured by an electrogoniometer connected to an A/D converter positioned on the left leg, where 180° was equivalent to full extension. Angles, EMG, and strength were recorded synchronously. The angles were measured to ensure that participants were in the correct position during the three-time points of the lift-off, mid-pull, and lock-out at approximately 95°, 126°, and 180°, respectively. For each key position, the participant performed the traction test isometrically with maximum force (The test protocol is described in the next item). During the test, the EMG activity of each muscle was evaluated in the three key positions. After data acquisition, the activity of each muscle was compared with itself for the three key positions.

### 4.3. Isometric Strength

**Figure 3:**
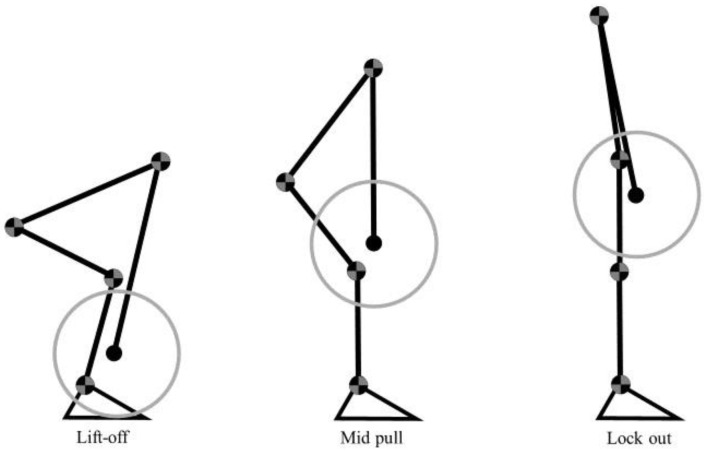
Illustration of the 3 DL positions analyzed: lift-off, mid-pull, and lock-out.

Participants were positioned on a metal platform with the barbell without plates attached to a chain. The participants were positioned on the platform and performed the familiarization of the movement by pulling the barbell three times with submaximal strength to adapt to body positioning. The barbell height for each test position corresponded to the three main DL positions of a concentric phase [16]. In the first position (lift-off), the barbell was positioned approximately 22.5 cm from the ground to correspond to the position of the barbell at the beginning of the movement at a height corresponding to the diameter of a plate. In the second position (mid-pull), the bar was positioned immediately above the patella. The third position (lock-out) corresponded to the triple extension of the knee, hip, and trunk with the barbell positioned above the midline of the thigh. Figure 3 shows the three positions used for analysis.

The order of each position was performed randomly. A pre-tension in the chain was allowed to avoid little or no vertical acceleration during traction. Participants were instructed to pull the bar and maintain the maximum effort for 4 s in each position with a 2–3 s rest interval and approximately 5 min of rest between each position [16]. The following strategies were adopted to reduce the margin of error in the tests: (a) standardized instructions were provided before the test so that the evaluated participant was aware of the entire routine that involved data collection; (b) the evaluated participant was instructed on the technique of performing the exercise; (c) the evaluator was aware of the position adopted by the practitioner at the time of the test, as small variations in the positioning of the joints involved in the movement could trigger other muscles, leading to erroneous interpretations; and (d) verbal stimuli were performed to maintain a high level of motivation. The suggested instructions were for the movement to be “as firm and fast as possible.” A load cell with a maximum reading of 200 kg with an acquisition frequency of 1000 Hz coupled to the same A/D converter was used to measure the pulling strength in each position. Strength data were analyzed using DasyLab v.11 software (National Instruments, Dublin, Ireland). The strength-time curves were smoothed using a fourth-order 10 Hz Butterworth low-pass filter [25] implemented in the software. The load cell was fixed to a metal base at one end and the other end was secured to the barbell by means of a chain. An official Olympic barbell weighing 20 kg was used. Strength was relativized by body mass and the maximum strength value of the three actions was used for the analysis.

### 4.4. Electromyographic Data

Signal acquisition was recorded by a SAS1000 V8 A/D converter (EMGSystem do Brasil, São José dos Campos, Brazil) connected to a computer. Medi-Trace 2000 silver chloride surface electrodes (Foam Graphic Controls Corporation, Gananoque, ON, Canada) with a bipolar configuration were used. The acquisition frequency for electromyographic data was 1000 Hz. In the locations corresponding to the analyzed muscles was shaving, skin abrasion using fine sandpaper, and cleaning of the area with 70% isopropyl alcohol according to the recommendations of SENIAM (Surface Electromyography for the Non-Invasive Assessment of Muscles) [26] available at www.seniam.org (accessed on 29 December 2022). The electrode location followed the standardization proposed by [26,27]. The electrodes were then applied with a center-to-center interelectrode distance of 30 mm. Skin impedance was verified with an ohmmeter attached to the connection socket of each pair of electrodes, being acceptable for the study. The selected muscles and their respective positions were: (1) RF muscle; the electrodes were positioned at 50% of the line of the anterior superior iliac spine to the superior part of the patella; (2) BF long head muscle; the electrodes were positioned at 50% of the line between the ischial tuberosity and the lateral epicondyle of the tibia; (3) ERE muscle; the electrodes were positioned two centimeters laterally to the spinous process of lumbar vertebra 1 (L1); (4) GAL muscle; the electrodes were positioned 1/3 between the fibular line and the heel. The reference electrode was positioned on the individual’s 7th cervical vertebra. In our study, we considered biarticular muscles that cross more than one joint, being able to move these joints, generating different actions. Thus, although it moves several vertebrae, we consider the ERE a uniarticular muscle, as it only performs extension in the lumbar region and keeps the trunk in the correct position throughout the movement, because of its importance ERE was analyzed.

**Figure 4:**
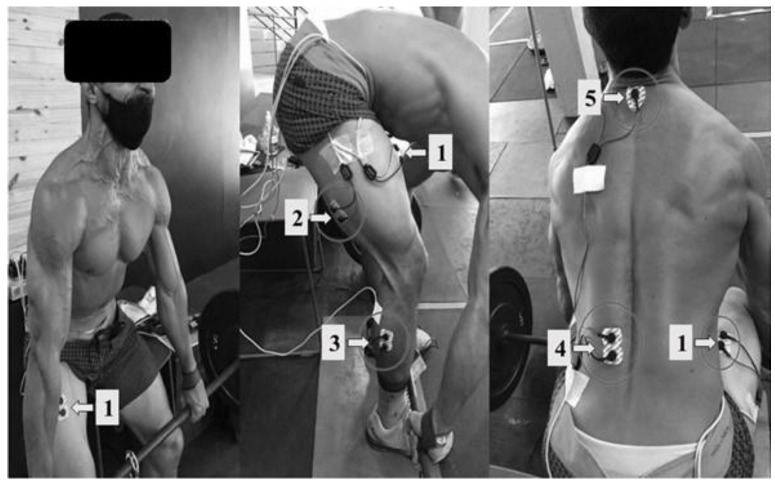
Evaluation of a participant and electrode placements: (1) rectus femoris muscle, (2) biceps femoris muscle, (3) lateral gastrocnemius muscle, (4) erector spinae muscle, and (5) reference electrode.

Figure 4 shows the placement of electrodes. Signals were measured during maximal isometric voluntary contractions (MIVC). After the acquisition, the signals were stored and treated with a 20–500 Hz bandpass filter and then converted into root mean square (RMS) by a routine of DasyLab v.11 (National Instruments, Dublin, Ireland). The maximal EMG value for each attempt of each muscle in the 3 positions was calculated, totaling 9 attempts per muscle. The EMG values in each participant for each muscle during each test were normalized as a percentage of the highest EMG value produced by that muscle [28]. EMG data were expressed as a percentage of maximal EMG amplitude produced by the muscle and referred to as the percentage of maximal isometric voluntary action (% MVC) [29].

### 4.5. Statistical Analysis

All data are shown as mean ± standard deviation. Previously, one-way ANOVA was performed to verify the differences between the pulling strength for each position with Bonferroni posthoc. Two-way ANOVA was performed with repeated measures by the general linear model, with muscles as fixed factors and positions as random factors, used for each dependent variable of interest, with a Bonferroni correction, where significant main effects were detected.

The sphericity was verified by Mauchly’s test. The magnitude of differences was calculated by the effect size η^2^ (partial eta square) and classified as small (0.0–0.25), medium (0.25–0.40), and large (above 0.40). The data were analyzed using SPSS 18.0 software (SPSS, Inc., Chicago, IL, USA) at the 5% significance level.

## Data Availability

The data presented in this study are available on request from the corresponding author.
